# Antipyretic and hepatoprotective potential of *Tinospora crispa* and investigation of possible lead compounds through in silico approaches

**DOI:** 10.1002/fsn3.1339

**Published:** 2019-12-23

**Authors:** Ahmed Rakib, Shahriar Ahmed, Md. Ashiqul Islam, Abdul Haye, S. M. Naim Uddin, Mir Muhammad Nasir Uddin, Mohammed Kamrul Hossain, Arkajyoti Paul, Talha Bin Emran

**Affiliations:** ^1^ Department of Pharmacy Faculty of Biological Science University of Chittagong Chittagong Bangladesh; ^2^ Department of Forensic Medicine University of Science and Technology Chittagong Chittagong Bangladesh; ^3^ Drug Discovery GUSTO A Research Group Chittagong Bangladesh; ^4^ Department of Microbiology Jagannath University Dhaka Bangladesh; ^5^ Department of Pharmacy BGC Trust University Bangladesh Chittagong Bangladesh

**Keywords:** antipyretic, CCl_4_‐induced hepatotoxicity, hepatoprotective, in silico studies, PASS prediction, *Tinospora crispa*, yeast‐induced pyrexia

## Abstract

This research describes an investigation of the antipyretic and hepatoprotective properties of both a crude organic extract and various subfractions of the ethnomedicinal plant *Tinospora crispa*, using appropriate animal models. In an attempt to identify potential lead hepatoprotective compounds, in silico experiments were utilized. Antipyretic activity was assessed via the Brewer's yeast‐induced pyrexia method, while hepatoprotective effects were evaluated in a carbon tetrachloride (CCl_4_)‐induced animal model. A computer‐aided prediction of activity spectra for substances (PASS) model was applied to a selection of documented phytoconstituents, with the aim of identifying those compounds with most promising hepatoprotective effects. Results were analyzed using Molinspiration software. Our results showed that both the methanol extract (METC) and various subfractions (pet ether, PEFTC; *n*‐hexane, NHFTC; and chloroform, CFTC) significantly (*p* < .05) reduced pyrexia in a dose‐dependent manner. In CCl_4_‐induced hepatotoxicity studies, METC ameliorated elevated hepatic markers including serum alanine amino transferase (ALT), aspartate amino transferase (AST), alkaline phosphatase (ALP), and total bilirubin. Malondialdehyde (MDA) levels were significantly reduced, while superoxide dismutase (SOD) levels were significantly increased. Among a selection of metabolites of *T. crispa*, genkwanin was found to be the most potent hepatoprotective constituent using PASS predictive models. These results demonstrate that both the methanolic extract of *T. crispa* and those fractions containing genkwanin may offer promise in reducing pyrexia and as a source of potential hepatoprotective agents.

## Graphical abstract

Methanol extract and its soluble fractions of *Tinospora crispa* exerted significant antipyretic activity and methanol extract is also responsible for hepatoprotective activity in vivo and in silico studies revealed that *T. crispa* possesses potential lead compounds for hepatoprotective activity.

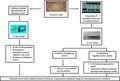

## 1 INTRODUCTION

Plants represent a vast source of phytochemicals, some of which may potentially be applied as natural therapeutic agents to treat various diseases. Bioactive phytoconstituents include alkaloids, tannins, vitamins, nucleosides, terpenoids, and sterols, which exhibit diverse pharmacological responses (Rice‐evans, Miller, Bolwell, Bramley, & Pridham, 1995). Within traditional medicine systems, plants are used for diverse reasons; among them, for hepatoprotective purposes and to treat fevers (Singhal & Gupta, 2012).

Pyrexia is a general clinical sign designated by the rise of body temperature beyond the normal range. Through this process, the body creates a suitable milieu for natural defense mechanisms, to facilitate repair of damaged tissue or to render infectious agents nonviable. Infected or damaged tissues produce different inflammatory mediators (cytokines including interleukin 1β, interferons α and β, TNF‐α), increasing the synthesis of prostaglandin E2 (PGE2) in the hypothalamus which causes the rise of body temperature (Khan, Rahman, & Islam, 2008). Almost all current antipyretic drugs block the synthesis of PGE2 via inhibition of the enzyme cyclooxygenase‐2 (COX‐2). Most of these therapeutic agents bind irreversibly with the enzyme COX‐2. Therefore, these synthetic agents are potentially toxic to the cortex of the brain, to muscles of the heart, to liver cells, and to the glomeruli. In contrast, it has been asserted that natural COX‐2 inhibitors have relatively less adverse effects (Emran, Dash, Uddin, & Rahman, 2018; Luo, He, & Bohlin, 2005).

The liver is the dominant organ associated with the metabolism of several types of xenobiotics. Biochemical events involved in metabolic processes include oxidation, reduction, hydrolysis, hydroxylation, sulfonation, acylation, and conjugation. Adverse changes in these metabolic functions are associated with the deterioration of hepatic parenchymal cells and hepatic injury (Wolf, 1999). Hepatic injury can be prompted by several infectious organisms, for example, viruses and by various hepatotoxins including CCl_4_, acetaminophen, thioacetamide, and ethanol, which are primarily metabolized by cytochrome P450 enzymes (Feijóo et al., 2010). Among these noxious agents, CCl_4_ is commonly used to induce hepatotoxicity in animal models, and also in the investigation of the role, lipid peroxidation plays in liver injury.

An example of a plant used in traditional medicine to treat febrile illness is *Tinospora crispa *Miers., a member of the family Menispermaceae. It is locally known as gulancha and is a lofty, woody, entirely glabrous climber (Yusuf, Chowdhury, Wahab, & Begum, 1994). The plant is indigenous to Eastern China, Bangladesh, India, Thailand, and Malaysia. Various phytochemicals, including alkaloids, lignans, triterpenes, nucleosides, and sterols, are known to be present in the plant* *(Ahmad, Jantan, & Bukhari, 2016). Previous research has shown that the methanol extract of the plant has significant antioxidant properties (Ahmad et al., 2016), while other species of the Menispermaceae such as *Tinospora cordifolia* and *Tinospora sinensis* have also been shown to possess significant hepatoprotective activities (Bishayi, Roychowdhury, Ghosh, & Sengupta, 2002; Nagarkar et al., 2013). Conversely, other workers have suggested that the stem juice of *T. crispa* possesses significant hepatotoxicity (Huang, Tu, Wang, & Huang, 2019). Various compounds, including furanoditerpenoids, have been implicated as responsible for this documented toxicity (Fukuda, Yonemitsu, & Kimura, 1993; Stickel, Patsenker, & Schuppan, 2005). These published reports are important when one considers the ethnobotanical role and uses of *T. crispa*. A decoction of the plant is employed for a variety of uses, primarily as a tonic and febrifuge, with the stem also given as a blood purifier and also to treat stomach disorders in the Khagrachari region of Bangladesh (Yusuf et al., 1994), while the tribal people of the Lawachara National Park in Mowlavi Bazar, Bangladesh, use the plant to alleviate abdominal pains and use an infusion of the plant as in the treatment of diabetes. Many variables may impact the bioactivity and safety of such remedies; among these, the nature of the prepared extracts and their posology. Moreover, the plant contains a plethora of phytoconstituents (Ahmad et al., 2016).

Therefore, in this present study, in an attempt to rationalize its indigenous use as a febrifuge, we sought to assess the antipyretic activity of both the methanol extract of *T. crispa *(METC) and various subfractions, namely the *n*‐hexane (NHFTC), chloroform (CFTC), and pet ether fractions (PEFTC). Furthermore, to explore the effects of METC and selected phytoconstituents on hepatic function, we investigated hepatoprotective activity using both a CCl_4_‐induced hepatotoxicity model and in silico* *analysis.

## 2 METHODS AND MATERIALS

### 2.1 Collection of plant materials

The whole plant of *T. crispa *was collected at the mature stage from the Lawachara National Park, Moulavi Bazar, Bangladesh, on January 2018. The plant was authenticated by Dr. Shaikh Bokhtear Uddin, Taxonomist and Professor, Department of Botany, University of Chittagong, Chittagong‐4331, Bangladesh. The plant parts were chopped into small pieces, washed thoroughly with water, and then dried in the shade at 21–30°C for 15 days. Later, the materials were dried in an oven at a low temperature to facilitate grinding. Afterward, the plant fragments were crushed using a mechanical grinder (Moulinex three‐in‐one grinder, China) and then passed through a size 60 mesh screen to obtain a fine powder. Finally, the powder was stored in an air‐tight container for future use.

### 2.2 Preparation of sample

The fine powder of the whole plant of *T. crispa* (600 g) was put in a clean Erlenmeyer flask (5 L) and soaked in 4 L of methanol for 15 days at room temperature with occasional shaking and stirring. The mixture was first filtered through a cotton plug, followed by a Whatman no. 1 filter paper. The filtrate was evaporated to dryness in a Heidorph rotary evaporator at 45°C, to obtain a concentrated extract. The semi‐solid extract was then air‐dried to obtain a solid residue. From this methanolic extract (METC), three subfractions were prepared, using sequential extraction with pet ether (PEFTC), chloroform (CFTC), and *n*‐hexane (NHFTC). All test samples, extracts, and standards for in vivo testing were formulated in normal saline using dimethyl sulfoxide (DMSO) and Tween 80 as cosolvents.

### 2.3 Chemicals and reagents

Methanol, pet ether, *n*‐hexane, chloroform, and CCl_4_ were purchased from Merck; paracetamol and silymarin were from Square Pharmaceuticals Limited. Normal saline solution (0.9% NaCl) was obtained from Orion Infusion Ltd. DMSO and Tween 80 were from BDH Chemicals, and the rest of the chemicals used were of analytical grade and from either BDH Fluka Chemie GmbH or Merck.

### 2.4 Experimental animals

Swiss albino mice (weighing 25–30 g, aged 4–5 weeks) and albino Wistar rats (weighing 150–200 g) and of either sex were used during the study. They were collected from the Animal Laboratory, Jahangirnagar University, Dhaka, Bangladesh. The animals were kept in groups of five in controlled laboratory conditions (12‐hr dark/12‐hr light cycle; temperature 25 ± 2°C) for 7 days for acclimatization. The animals were given standard feed and water *ad libitium*. The animals were fasted overnight and were weighed before the experiments. The care and handling of animals were in accordance with Institutional Ethical Guidelines of Faculty of Biological Sciences, University of Chittagong (Pharmacol/DPH/UC/01, 2018).

### 2.5 Acute toxicity studies

An acute oral toxicity study was performed according to the OECD guidelines for the testing of chemicals, Test No. 423 (OECD, 2001; Acute oral toxicity‐acute toxic class method). A total of five animals used in the toxicity study received a single oral dose of either 500 mg/kg b.w., 1,000 mg/kg b.w., 1,500 mg/kg b.w., or 2000 mg/kg b.w. of *T. crispa *extract. Following dosing, food was withheld for 3 to 4 hr. The individual animals were closely observed during the first 30 min after dosing, periodically for the first 24 hr, after that for 3 days to record any delayed toxicity. Other changes, including in skin and fur, eyes and mucous membranes, respiratory and circulatory rate or autonomic and CNS function, were observed. The median lethal dose was used (LD50 > 2.0 g/kg) to calculate the effective therapeutic dose, as described by Zaoui et al., 2002.

### 2.6 Antipyretic testing

The antipyretic activity of METC and its various subfractions were evaluated in Swiss albino mice (25–30 g) of either sex by inducing pyrexia via the administration of Brewer's yeast suspension. The animals were divided into ten groups, each having five mice. Normal body temperature was recorded with the help of a digital thermometer, with pyrexia induced thereafter by the administration of a 20% aqueous suspension of Brewer's yeast (10 ml/kg, s.c.). All groups were fasted overnight but allowed free access to drinking water, and after 18h, the rectal temperature of each mouse was recorded. An induction of pyrexia was confirmed by the elevation of temperature more than 0.5ºC (Kang et al., 2008). Group I received normal saline (10 ml/kg) as a negative control, group II received paracetamol (150 mg/kg) as a standard drug while the remaining groups III‐X received METC, CFTC, PEFTC, or NHFTC, at 200 mg/kg or 400 mg/kg, respectively. After the administration of control, standard, and test samples, the rectal temperature was recorded at 1 hr, 2 hr, 3 hr, and 4 hr.

### 2.7 In vivo hepatoprotective activity

A total of 30 (thirty) rats were divided into six groups of five rats each. Group I served as a normal control group and received only the vehicle (1 ml kg^‐1^ day^‐1^ of 1% CMC, p.o.). Group II served as the positive control and received CCl_4_ 1 ml/kg (1:1 of CCl_4_ in olive oil) i.p. once daily for 7 days. Group III served as a standard group and received CCl_4_ 1 ml/kg (1:1 of CCl_4_ in olive oil) i.p. and silymarin 100 mg/kg orally (p.o.) for 7 days. Groups IV, V, and VI were administered METC at 100, 200, or 400 mg/kg body weight p.o., respectively, and a dose of 1 ml/kg i.p. of CCl_4_ (1:1 of CCl_4_ in olive oil) for 7 days. All rats were sacrificed by cervical dislocation 24 hr after the last treatment. Just before sacrifice, blood was collected from the retro‐orbital sinus plexus under mild ether anesthesia. The collected blood was allowed to clot, and the serum was separated at 1,372 *g* for 15 min prior to carrying out further biochemical investigations. A section of each liver was separated and used for biochemical studies (Al Mahmud et al., 2016; Bulbul et al., 2019; Chandan et al., Domitrović, Jakovac, Milin, & Radošević‐Stašić, 2009).

### 2.8 Measurement of serum biochemical parameters

The activities of serum glutamic‐pyruvic transaminase (SGPT), serum glutamic oxaloacetate transaminase (SGOT), alkaline phosphatase (ALP), and total bilirubin (TB) were determined using the Hitachi 912 clinical chemistry automatic analyzer (Roche Diagnostic GmbH, Mannheim, Germany).

### 2.9 Assessment of lipid peroxidation and superoxide dismutase (SOD)

The excised livers were perfused with chilled normal saline to remove any blood cells. They were then cut into small pieces, placed in 0.1M phosphate buffer (pH 7.4), and homogenized. The homogenate was centrifuged at 1,008 *g* for 15 min, and the supernatant was collected in an Eppendorf tube. The supernatant was again centrifuged at 16,128 *g* for 30 min. The final supernatant was used for the determination of MDA as a lipid peroxidation marker, while SOD was also assayed (Smyth et al., 2008; Wu, Li, Wen, & Li, 2007; Zeashan, Amresh, Singh, & Rao, 2008).

### 2.10 In silico experiment to predict the activity spectra for substances (PASS)

**Figure 1:**
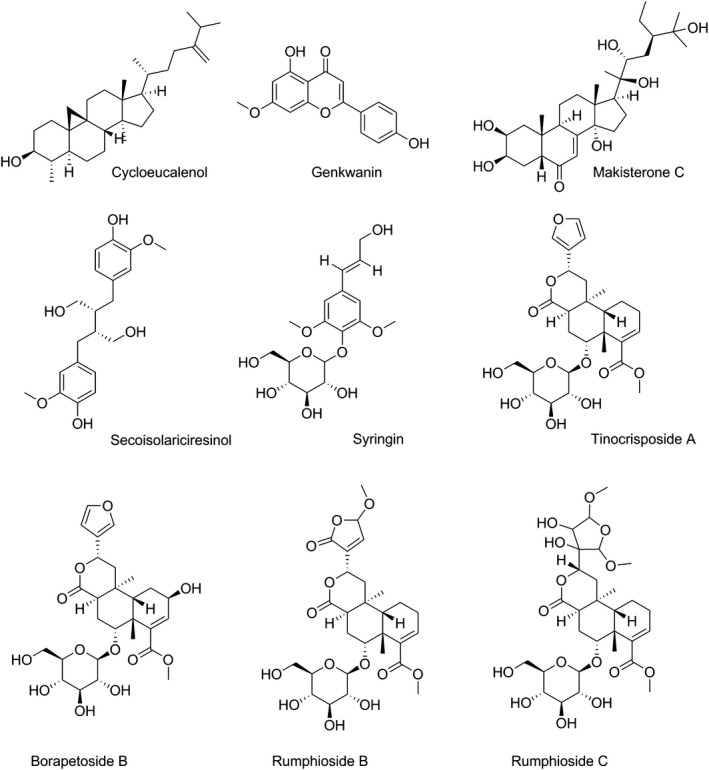
Structures of all (nine) selected phytoconstituents

Structures of all (nine) selected phytoconstituents, namely cycloeucalenol, genkwanin, makisterone C, secoisolariciresinol, syringin, tinocrisposide A, borapetoside B, rumphioside B, and rumphioside C (see Figure 1) (Ahmad et al., 2016; Cavin, Hostettmann, Dyatmyko, & Potterat, 1998; Chung, 2011; Kongkathip et al., 2002; Martin, Ohtani, Kasai, & Yamasaki, 1996; Pachaly, Adnan, & Will, 1992; Umi Kalsom & Noor, 1995) were obtained from the PubChem database and subjected for evaluation of their hepatoprotective activities using the PASS program. This experiment predicts a compound's activity spectrum as probable activity (P_a_) or probable inactivity (P_i_) (Ahmed et al., 2019; Mojumdar et al., 2016). The results are based on a structure–activity relationship (SAR) analysis of the training set, which consists of more than 180,000 compounds showing greater than 3678 types of biological activities (Parasuraman, 2011). The values of P_a_ and P_i_ lie within the range 0.000 to 1.000. When P_a_ is greater than P_i_, the compound is thought to be experimentally active. P_a_ > 0.7 indicates the probability of pharmacological potential is rich with values 0.5 < P_a_ <0.7 reflecting considerable pharmacological effects experimentally. P_a_ < 0.5 shows less pharmacological activity (Goel, Singh, Lagunin, & Poroikov, 2011; Khurana, Ishar, Gajbhiye, & Goel, 2011).

### 2.11 Drug‐likeness calculation and prediction of biological activity

The targeted compounds were assessed for their drug‐likeness characteristics based on the Lipinski rule of five and were also assessed for potential bioactivity by calculating their activity scores as GPCR ligands, ion channel modulators, kinase inhibitors, nuclear receptor inhibitors, and enzyme inhibitors. All the parameters were checked with the aid of the software Molinspiration (http://www.molinspiration.com) (Verma, 2012). Calculated drug‐likeness scores of each compound were compared with the specific activity of each compound and were compared with the standard drug (silymarin).

### 2.12 Statistical analysis

Data were expressed as mean ± SEM (Standard error of the mean) of five animals. For statistical analysis, analysis of variance (ANOVA) was followed by post hoc Dunnett's test for multiple comparisons. The outcomes were considered to be significant at the *p* < .05 level. The statistical analysis was carried out using either the statistical software package for social science (SPSS, version 20.0, IBM Corporation), Prism version 7.0a (GraphPad Software Inc.), or Microsoft^®^ Excel.

## 3 RESULTS

### 3.1 Acute toxicity studies

All the extracts and fractions of the plant *T.* *crispa* did not cause any mortality up to 2000 mg/kg dose level. Therefore, 100, 200, and 400 mg/kg doses were selected for further experiments.

### 3.2 Antipyretic test

Statistical analysis revealed that both the methanol extract and subfractions of *T. crispa* showed significant antipyretic activity. METC and PEFTC showed significant results at 2, 3, and 4 hr, using a 400 mg/kg dose. On the other hand, NHFTC exhibited extremely significant results at all time points using the 400 mg/kg dose. The inhibition was dose‐dependent. The effect of the extract and fractions on the rectal temperature in mice is presented in Table 1.

**Table 1:** Antipyretic activity of extract and fractions of *Tinospora crispa* by Brewer's yeast‐induced pyrexia method

Group	Initial rectal temperature before yeast injection (°F)	Rectal temperature at yeast injection and after the administration of sample (°F)				
		0 hr	1st hour	2nd hour	3rd hour	4th hour
Control (Distilled water)	99.27 ± 0.29	100.27 ± 0.27	100.27 ± 0.22	100.3 ± 0.20	100.23 ± 0.19	100.3 ± 0.15
Standard (Paracetamol 100 mg/kg)	98.56 ± 0.10	100.56 ± 0.33	99.82 ± 0.12	99.20 ± 0.12**	98.86 ± 0.05**	98.32 ± 0.10**
METC (200 mg/kg)	98.87 ± 0.15	100.33 ± 0.89	100.13 ± 0.67*	99.87 ± 0.67*	99.73 ± 0.09**	99.52 ± 0.15*
METC (400 mg/kg)	99.18 ± 0.89	99.83 ± 0.09	99.17 ± 0.09**	98.93 ± 0.67***	98.7 ± 0.06***	98.6 ± 0.11***
PEFTC (200 mg/kg)	99.85 ± 0.15	100.73 ± 0.19	100.47 ± 0.17	100.07 ± 0.12	99.83 ± 0.12	99.78 ± 0.09
PEFTC (400 mg/kg)	99.43 ± 0.20	100.7 ± 0.12	99.4 ± 0.10	99.10 ± 0.17***	98.9 ± 0.15***	98.87 ± 0.23***
CFTC (200 mg/kg)	98.76 ± 0.18	100.07 ± 0.18	99.83 ± 0.89	99.27 ± 0.67***	99.10 ± 0.15***	98.80 ± 0.20***
CFTC (400 mg/kg)	98.80 ± 0.12	100.31 ± 0.15	99.33 ± 0.24**	98.83 ± 0.13***	98.57 ± 0.12***	98.37 ± 0.12***
NHFTC (200 mg/kg)	98.60 ± 0.12	99.80 ± 0.06	99.77 ± 0.06*	99.57 ± 0.09*	99.34 ± 0.03**	99.21 ± 0.06***
NHFTC (400 mg/kg)	98.73 ± 0.12	100.01 ± 0.05	98.8 ± 0.17***	98.5 ± 0.10***	98.07 ± 0.09***	98.03 ± 0.20***

Note

Values are expressed as mean ± *SEM* or percentage (*n* = 5). The data were analyzed by one‐way ANOVA followed by Dunnett's test. Asterisks indicated statistically significant values from control.

Abbreviations: CFTC, chloroform fraction of *T. crispa*; METC, methanolic extract of *T. crispa;* PEFTC, pet ether fraction of *T. crispa*.

*p < .05, **p < .01, and ***p < .001 compared with control.

### 3.3 In vivo hepatoprotective activity

#### 3.3.1 Effect of METC on the measurement of serum biochemical parameters

The hepatoprotective effects of METC on serum biochemical parameters in CCl_4_‐intoxicated rats are shown in Table 2. Rats treated with CCl_4_ (group II) showed a significant increase in serum SGPT, SGOT, ALP, and total bilirubin levels compared to control animals (group I). Pretreatment with METC at 100, 200, and 400 mg/kg for 7 days (groups IV, V, and VI) showed significant hepatoprotection in terms of serum SGPT, SGOT, ALP, and total bilirubin levels compared to the positive control group (group II), with highest administered doses achieving reductions comparable to those of the standard drug (silymarin).

**Table 2:** Effects of METC on serum biochemical parameters in CCl_4_‐intoxicated rats

Group	SGPT (U/L)	SGOT (U/L)	ALP (U/L)	Serum Bilirubin (mg/dl)	MDA	SOD
Control	180.33 ± 0.88	65.33 ± 0.88	200.66 ± 1.20	0.50 ± 0.01	100.67 ± 1.20	10.46 ± 0.53
CCl_4_ (2 ml/kg)	620 ± 0.57***	130.33 ± 0.88***	550.33 ± 0.81***	0.98 ± 0.01***	130.33 ± 0.77***	4.33 ± 0.08***
Silymarin (100 mg/kg)	240.33 ± 0.81***	71 ± 0.57**	200.66 ± 1.20***	0.43 ± 0.01***	111 ± 0.56**	10.33 ± 0.87***
METC (100 mg/kg)	431 ± 0.55***	86.33 ± 0.83***	262 ± 1.52**	0.67 ± 0.01***	130.33 ± 0.89***	7.66 ± 0.76**
METC (200 mg/kg)	321.34 ± 0.89***	77 ± 0.56***	211.33 ± 0.85***	0.7 ± 0.05***	120.65 ± 1.20***	6.5 ± 0.06***
METC (400 mg/kg)	251.33 ± 0.68***	66.33 ± 0.77***	210.66 ± 1.21***	0.57 ± 0.02***	130.66 ± 1.21***	8.26 ± 0.10***

Note

Values are expressed as mean ± *SEM* or percentage (*n* = 5). The data were analyzed by one‐way ANOVA followed by Dunnett's test. Asterisks indicated statistically significant values from control.

Abbreviations: CFTC, chloroform fraction of *Tinospora crispa*; METC, methanolic extract of *T. crispa;* PEFTC, pet ether fraction of *T. crispa.*

*p < .05, **p < .01 and ***p < .001 compared with control.

#### 3.3.2 Effect of METC on MDA and SOD levels

Lipid peroxidation was increased in the toxic control group, as revealed by elevated MDA levels when compared with the normal control group. Pretreatment with METC at 100, 200, and 400 mg/kg significantly decreased the MDA levels, which were almost similar to those of rats receiving the standard drug silymarin. Levels of the antioxidant enzyme, SOD, were increased considerably in METC treated groups. The extract, at a dose of 400 mg/kg, demonstrated maximum hepatoprotection, as shown in Table 2.

### 3.4 In silico PASS prediction

Nine compounds previously isolated from the plant, namely cycloeucalenol, genkwanin, makisterone C, secoisolariciresinol, syringin, tinocrisposide A, borapetoside B, rumphioside B, and rumphioside C were analyzed by the PASS program for their hepatoprotective effects. The results obtained by the PASS prediction are shown in Table 3.

**Table 3:** PASS prediction of cycloeucalenol, genkwanin, makisterone C, secoisolariciresinol, syringin, tinocrisposide A, borapetoside B, rumphioside B, rumphioside C for hepatoprotective activity

Compounds	Pass prediction of hepatoprotective activity	
	Pa	Pi
Cycloeucalenol	0.703	0.007
Genkwanin	0.633	0.010
Makisterone C	0.457	0.024
Secoisolariciresinol	0.426	0.028
Syringin	0.846	0.003
Tinocrisposide A	0.924	0.002
Borapetoside B	0.903	0.002
Rumphioside B	0.889	0.003
Rumphioside C	0.875	0.003

### 3.5 Drug‐likeness calculation based on Lipinski's rule of five

With the help of Molinspiration software, we calculated different properties of the target compounds. The compounds showed different drug‐likeness scores and were compared with the standard drug silymarin. The results are shown in Table 4.

**Table 4:** Properties of compounds at different parameters based on Lipinski's rule of five

Compounds	milog P	TPSA	*n* atoms	MW	nON	nOHNH	*n* violations	*n* rotb	Volume
Cycloeucalenol	7.62	20.23	31	426.73	1	1	1	5	462.17
Genkwanin	3	79.9	21	284.27	5	2	0	2	241.58
Syringin	−0.66	138.08	26	372.37	9	5	0	7	327.051
Tinocrisposide A	1.54	165.13	38	536.57	11	4	2	6	473.79
Borapetoside B	0.39	185.36	39	552.57	12	5	2	6	481.83
Rumphioside B	1.14	187.53	41	582.6	13	4	2	7	507.73
Rumphioside C	−0.45	220.15	44	632.66	15	6	3	8	553.07
Silymarin	1.47	155.15	35	482.44	10	5	0	4	400.86

### 3.6 Prediction of biological activity

Eight selected compounds of the plant *T. crispa* were subject to biological activity calculations with the help of Molinspiration software and compared with standard drug silymarin. The results are shown in Table 5.

**Table 5:** Biological activity of compounds on different parameters

Compounds	GPCR ligand	Ion channel inhibitor	Kinase inhibitor	Nuclear receptor ligand	Protease inhibitor	Enzyme inhibitor
Cycloeucalenol	0.14	0.14	−0.37	0.92	0.1	0.61
Genkwanin	−0.08	−0.16	0.17	0.33	−0.25	0.2
Syringin	0.11	0.09	−0.08	0.09	−0.04	0.38
Tinocrisposide A	0.39	−0.04	−0.27	0.21	0.07	0.46
Borapetoside B	0.42	−0.01	−0.29	0.21	0.07	0.49
Rumphioside B	0.24	−0.27	−0.33	0	0.09	0.43
Rumphioside C	0.15	−0.41	−0.51	−0.13	0.14	0.29
Silymarin	0.07	−0.05	0.01	0.16	0.02	0.23

## 4 DISCUSSION

This study reveals the antipyretic effects of both METC and its subfractions, and discloses the hepatoprotective activity of METC and in silico hepatoprotective activity evaluation of *T. crispa.*

Subcutaneous injection of Brewer's yeast increases the production of prostaglandins that induce pyrexia. The Brewer's yeast‐induced pyrexia method is considered a convenient test for the screening of synthetic drugs as well as of plant phytochemicals for their antipyretic effects (Devi, Boominathan, & Mandal, 2003; Khan et al., 2009). Antipyretic activity may be exerted by inhibition of the synthesis of prostaglandin, which is possibly achieved via blockade of cyclooxygenase (COX) enzyme activity. Many mediators, such as tumor necrosis factor (TNF), interleukin (IL)‐1, IL‐6, interferons, collaborate in the elevation of temperature, and curbing the activity of some of these mediators is likely responsible for observed antipyretic effects (Rawlins & Postgrad, 1973). In rodents, the subcutaneous administration of cell wall products of *Saccharomyces cerevisiae* act as exogenous pyrogens, stimulating immune cells such as lymphocytes and macrophages. As a result, endogenous pyrogens are produced in the form of cytokines, which reach the hypothalamus through the circulation and elicit a change in body temperature (Miller, 2000). The administration of METC and its subfractions (PEFTC, NHFTC, CFTC) significantly decreased the rectal temperature of treated mice. This reduction in temperature may be due to the presence of pharmacologically active constituents in *T. crispa*, which interfere with the synthesis of prostaglandin. However, diverse biochemical events occur during the biosynthesis of prostaglandins and further work is necessary to determine the exact point in the biosynthetic pathway whereby the extract exerts its antipyretic effects.

The liver is mainly associated with the detoxification of different toxic chemicals and drugs, including most possible xenobiotics which are toxic in nature. CCl_4_ is a potent hepatotoxin and widely used in animal models for the assessment of hepatoprotective activity of various chemicals. Hepatic damage inflicted by CCl_4_ resembles the pathology of viral hepatitis (Ravikumar & Gnanadesigan, 2012; Shim et al., 2010). Cytochrome P450 enzymes metabolize CCl_4_, forming CCl_3_^•^ free radicals, which can react with oxygen to form the trichloromethyl peroxyl radical (CCl_3_O_2_^•^), which covalently binds to cellular macromolecules and biological membranes, causing lipid peroxidation. The peroxide products then signal the production and leakage of biomarkers like MDA. This whole cascade reaction ultimately causes loss of cellular integrity and liver damage (Dolai et al., 2012; Jain et al., 2011; Kiran, Raju, & Rao, 2012; Nirmala, Girija, Lakshman, & Divya, 2012; Ravikumar & Gnanadesigan, 2011; Sengupta, Sharma, & Chakraborty, 2011; Shim et al., 2010; Thabit, Al‐Moyed, Al‐Balushi, Hasson, & Sallam, 2012; Thirumalai, David, Therasa, & Elumalai, 2011; Zeashan et al., 2008). Lipid peroxidation is also a functional parameter of oxidative stress as well as other free radical damage that happens during the biological cascade. Consequently, antioxidant efficacy is often cited as a marker of hepatoprotection (Emran et al., 2015; Singhal & Gupta, 2012; Uddin et al., 2019).

SGPT, SGOT, and ALP are serum hepatobiliary enzymes present at higher concentrations in the liver. The trichloromethyl peroxyl radical (CCl_3_O_2_^•^) directly interacts with polyunsaturated fatty acids present in the endoplasmic reticulum (ER), causing the elevated production of biomarker enzymes such as SGPT, SGOT, ALP, and bilirubin (Hanafy, Aldawsari, Badr, Ibrahim, & Abdel‐Hady, 2016). Also, these biomarker enzymes are elevated by necrosis or hepatic damage, and they lead to elevated serum concentrations (Zeashan et al., 2008). Increased levels of SGPT, SGOT, and ALP in CCl_4_‐induced animals also reflect cellular damage and diminution of cell membrane function (Zeashan et al., 2008).

Lipid peroxidation is an autocatalytic process and may lead to cell death. During the lipid peroxidation process, several end products are formed, including malondialdehyde (MDA), formed during oxidative degeneration as a product of oxygen free radicals and accepted as an indicator of lipid peroxidation (Tukappa, Londonkar, Nayaka, & Kumar, 2015). MDA levels are increased by the induction of CCl_4_, which increases the rate of lipid peroxidation, thus promoting hepatic tissue damage (Huang et al., 2010).

Antioxidants usually prevent the lipid peroxidation reaction in normal circumstances. However, in CCl_4_‐induced animals, as a result of the hepatic tissue damage due to a higher rate of lipid peroxidation, there is simultaneous reduction in the estimation of SOD which suggests the collapse of antioxidant defense mechanisms in inhibiting the damage done by peroxidation.

The rise in the levels of SGPT, SGOT, and ALP enzymes in CCl_4_‐treated animals confirms the hepatic damage and explains the presence of the breakdown product heme in red blood cells, thus elevating the levels of bilirubin, which is a pathophysiological and clinical indicator of hepatic tissue necrosis.

From the results of the present study, METC significantly reduces SGPT, SGOT, ALP, and bilirubin levels in the serum as compared to the standard drug silymarin, in a dose‐dependent manner, and decreases the amount of the leakage of intracellular biomarker enzymes by stabilizing the hepatic cellular membrane. The significant decrease in MDA levels with an increase in the dose of METC suggests that its predominant mechanism of hepatoprotection may be through decreasing lipid peroxidation. Additionally, the rise in SOD levels also suggests that METC contributes to the repair of antioxidant defense systems. METC has significant documented antioxidant properties (Ahmad et al., 2016). Furthermore, other Menispermaceae plants such as *Tinospora cordifolia* and *Tinospora sinensis* have potential hepatoprotective effects (Bishayi et al., 2002; Nagarkar et al., 2013).

Screening of hepatoprotective properties of different phytochemicals present in *T. crispa* was performed using the PASS program. Among the selected compounds, cycloeucalenol, genkwanin, syringin, tinocrisposide A, borapetoside B, rumphioside B, and rumphioside C exhibited the highest P_a_ values for hepatoprotective activity, with tinocrisposide A showing the highest individual P_a_ value of 0.924.

Considering Lipinski's rule of five, compounds may be considered more favorably as drug candidates if they have a partition coefficient of less than 5, a total polar surface area within 140, have less than 10 hydrogen bond acceptors and 5 hydrogen bond donors, and have a molecular weight within 500 Daltons (Verma, Masoodi, & Ahmed, 2012). Among the screened phytoconstituents of *T. crispa*, only genkwanin follows Lipinski's rule of five. However, Lipinski's rule is routinely violated by many useful natural product drugs.

Based on the known activities of silymarin, including inhibition of proteases and kinases, we compared the selected compounds for comparable hepatoprotective activity. An increase in score signifies enhanced inhibition properties (Lalitha & Sivakamasundari, 2010). All compounds, except genkwanin, showed better inhibition of these enzymes compared to the standard silymarin, with cycloeucalenol exhibiting the best antihepatotoxic activity.

Importantly, as aforementioned, previous work has suggested that *T. crispa* may be hepatotoxic and therefore that consuming the stem juice of *T. crispa* could elevate the chance of liver toxicity (Langrand et al., 2014; Denis et al., 2007). This could result from the presence of intrinsic hepatotoxins or via metabolism to toxic entities. Conversely, in our experiments, PASS prediction and in silico analysis suggested that certain compounds from *T. crispa* may possess hepatoprotective properties. This is in alignment with previous research on such compounds, including cycloeucalenol, which are known for their hepatoprotective effects (Ismail & Choudhary, 2016). Moreover, syringin also exhibited hepatoprotective activities by demonstrating protection against LPS/D‐GalN‐induced FHF, thus inhibiting apoptosis of hepatic cells, and alleviating liver injury (Gong et al., 2014).

## 5 CONCLUSION

The methanol extract of *T. crispa* and its subfractions were investigated for in vivo antipyretic and hepatoprotective activities, while some known phytoconstituents were screened in silico. Our investigations showed that the methanol extract and its subfractions exerted significant antipyretic effects and that the methanol extract possessed potential hepatoprotective effects against CCl_4_‐induced toxicity in Wistar rats. Moreover, from the PASS prediction and in silico studies, it is likely that some of the screened compounds may be responsible for the observed hepatoprotective activity. *Tinospora crispa* and its extracts thus represent an interesting paradox, having the potential to exhibit hepatotoxic or hepatoprotective properties, and warrants further study.

## CONFLICT OF INTEREST

The authors declare that they have no competing interests.

## AUTHOR CONTRIBUTIONS

All the authors have accepted responsibility for the entire content of this submitted manuscript and approved the submission. Authors AR and SA collected the plant sample and prepared the extracts and fractions. AR, SA, MAI, AH, SMNU, MMNU, MKH, AP, and TBE carried out the study design, performed the experiments, data collection, data interpretation, manuscript preparation, statistical analysis. Authors SMNU, MMNU, MKH, and TBE designed and planned the studies and supervised the experiments.

## ETHICAL APPROVAL

The authors declare that there is no conflict of interest regarding the publication of this article. This study does not involve any human testing. The research described herein was performed on Swiss albino mice and albino Wistar rats of either sex. This study was performed in strict accordance with Institutional Ethical Guidelines of Faculty of Biological Sciences, University of Chittagong (Ref. No. Pharmacol/DPH/UC/01, 2018).
